# Health policy and facility factors influencing the effectiveness of medical assessments for people with disabilities in Hanoi, Vietnam: A qualitative study

**DOI:** 10.1097/MD.0000000000050922

**Published:** 2026-10-02

**Authors:** Dang Duc Nhu, Nguyen Ngoc Nghia, Nguyen Ngoc Thanh, Nguyen Thanh Trung, Phung Lam Toi, Nguyen Huynh Phuong Anh, Tran Thi Ly, Nguyen Trong Khoa, Duong Huy Luong, Tran Quoc Thang, Luong Thi Thu Ha, La Ngoc Quang, Phan Hoang Hiep, Tran Vuong The Vinh

**Affiliations:** a Faculty of Public Health, University of Medicine and Pharmacy, Vietnam National University at Hanoi, Hanoi, Vietnam; b Hanoi Medical Assessment Center, Hanoi, Vietnam; c Department of Medical Service Administration, Ministry of Health, Hanoi, Vietnam; d Faculty of Public Health, Phenikaa School of Medicine and Pharmacy, Phenikaa University, Hanoi, Vietnam; e Thang Long Institute of Medical and Pharmaceutical Research, Hanoi, Vietnam; f Department of Epidemiology, Faculty of Fundamental Sciences, Hanoi University of Public Health, Hanoi, Vietnam; g National Hospital of Endocrinology, Hanoi, Vietnam; h Department of Surgery, Hai Phong University of Medicine and Pharmacy, Hai Phong, Vietnam.

**Keywords:** administration, communication, disability assessment, health policy, infrastructure, people with disabilities

## Abstract

Disability assessment determines access to social and healthcare support, yet the operation of assessment pathways may be shaped by policy implementation and facility capacity. We conducted a qualitative descriptive study at the Hanoi Medical Assessment Center, Vietnam, using purposive sampling and semi-structured, in-depth interviews with 13 stakeholders, including people with disabilities, guardians, administrative and reception staff, district social-protection officials, medical examiners, and center leaders. Interviews were audio-recorded, transcribed verbatim, and analyzed using thematic analysis. Four interconnected themes were identified: administrative fragmentation and procedural burden, communication gaps and reliance on informal information channels, infrastructure constraints and system-level inefficiencies, and financial ambiguity and trust-related concerns. Participants described multilevel administrative handoffs and episodic dossier submission as sources of delay; official information was often difficult to interpret, leading users to rely on healthcare professionals; limited on-site diagnostic capacity required external referrals; and uncertainty about fees, insurance coverage, and the distinction between paying for assessment and qualifying for benefits undermined trust. These findings reflected experiences at 1 urban center and were not intended to estimate prevalence or represent disability assessment nationally. They identify context-specific service priorities for Hanoi, including clearer inter-level responsibilities, accessible and standardized guidance, better coordination of diagnostic services, and transparent communication of costs. Multicenter research, including rural and under-resourced settings, is needed before broader policy implications can be drawn.

## 1. Introduction

Accurate and accessible disability assessment is a gateway to social protection, healthcare, rehabilitation, and other disability-related entitlements. An estimated 1.3 billion people, approximately 16% of the global population, experience significant disability, and many continue to face inaccessible information and facilities, unaffordable transport and services, and fragmented support within health systems. Because assessment decisions determine eligibility and access, weaknesses in the assessment pathway may compound existing inequities rather than merely create administrative inconvenience.^[1,2]^

The performance of a disability assessment system depends not only on clinical criteria or examiner competence but also on how applicants move between health, administrative, and social-protection institutions. Clear eligibility information, consistent documentation requirements, timely referral and diagnostic capacity, coordination between levels of government, and transparent costs are therefore implementation requirements. Evidence from Vietnam has shown that people with disabilities (PWDs) may have incomplete knowledge of available entitlements and may encounter procedural and financial barriers when seeking social protection. Qualitative evidence from low- and middle-income countries (LMICs) likewise indicates that informational, attitudinal, physical, financial, and logistical barriers interact across the service pathway rather than operating in isolation.^[3,4]^

In LMICs, these challenges are often amplified by systemic constraints, including limited infrastructure, fragmented administrative systems, and inadequate communication channels.^[5]^ Studies in global health have highlighted that health policy design alone is insufficient to ensure effective implementation; instead, the interaction between policy, institutional capacity, and service delivery environments plays a critical role. In particular, qualitative evidence suggests that administrative complexity, unclear procedures, and limited awareness among beneficiaries can hinder access to disability-related services. Despite these insights, there remains a lack of in-depth qualitative research exploring how policy and facility-level factors interact to influence the effectiveness of disability assessment systems, especially in Southeast Asian contexts.^[6,7]^

Vietnam has made significant progress in developing a comprehensive policy framework to support PWDs, including regulations governing medical assessment for disability determination. However, the implementation of these policies in real-world settings, particularly at local levels, may face multiple challenges related to administrative coordination, infrastructure capacity, and communication strategies. To date, few studies have examined these issues from the perspectives of multiple stakeholders involved in the assessment process, including PWDs, healthcare providers, and administrative officials.^[3]^

Previous work has examined disability-targeted social protection and access barriers in Vietnam, but less is known about the day-to-day operation of a city-level medical assessment center and the handoffs between service users, clinical examiners, center staff, and district social-protection officials.^[3]^ International evidence also shows that similar barriers are highly context-dependent. A focused examination of 1 assessment pathway can therefore contribute implementation evidence while avoiding claims of national representativeness. Accordingly, this qualitative study explored how stakeholders experienced policy implementation and facility-level factors affecting medical assessment for PWDs at the Hanoi Medical Assessment Center.

## 2. Methods

### 2.1. Study design, ethics and setting

This qualitative descriptive study used semi-structured, in-depth interviews to explore policy and facility-level factors affecting medical assessment for PWDs. The study was approved by the Institutional Review Board of the University of Public Health, Hanoi, Vietnam (Institutional Review Board No.: 22124/HDDD). The study was conducted in accordance with the Declaration of Helsinki and complied with national regulations on biomedical research involving human participants in Vietnam. Informed consent was obtained from all participants prior to the interviews. The study was conducted at the Hanoi Medical Assessment Center, a city-level institution that receives cases from multiple districts and coordinates clinical and administrative components of disability assessment. Data were collected from October 2022 to October 2023. The study is reported in accordance with the Consolidated Criteria for Reporting Qualitative Research.^[8]^

### 2.2. Participants and sampling

Purposive sampling was used to obtain information-rich perspectives from different positions in the assessment pathway. Eligible participants were PWDs or guardians/legal representatives with direct experience of assessment at the center, and personnel who received, submitted, coordinated, performed, or supervised disability-assessment activities during the study period. Medical examiners were required to have at least 6 months of relevant assessment experience. Individuals were excluded if they did not consent to participate or lacked direct experience of the assessment pathway. The previous criterion concerning incomplete medical records was removed because medical-record completeness was not relevant to eligibility for this interview study. Recruitment sought variation by stakeholder role rather than statistical representativeness. Interviews continued until additional data no longer generated substantively new codes or themes relevant to the study aim. The final sample comprised 13 participants, which was judged adequate for the focused study question and the specific, information-rich stakeholder groups.^[9]^

### 2.3. Data collection

The research team developed a semi-structured interview guide from the study objectives and relevant disability-policy and health-systems literature. The guide covered administrative procedures, communication and access to information, infrastructure and diagnostic resources, financial issues, and recommendations for service improvement. Question wording and probes were adapted to participant role. The role-specific guides for PWDs/legal representatives, social-protection officials, and center leaders are provided as Supplementary Digital Content 1. Interviews were conducted face-to-face in a private room at the assessment center or the participant’s workplace and lasted approximately 30 to 60 minutes. Trained researchers with backgrounds in public health and clinical medicine and prior qualitative-research experience conducted the interviews. With permission, interviews were audio-recorded, and field notes documented context and nonverbal observations. Interviews were conducted in Vietnamese and transcribed verbatim in Vietnamese. Analysis was undertaken using the original-language transcripts; illustrative quotations were translated into English for publication.

### 2.4. Trustworthiness and researcher reflexivity

Credibility was supported by triangulating accounts across service users, family representatives, administrative personnel, medical examiners, district officials, and center leaders. Dependability was strengthened through a documented coding framework and an audit trail of analytical decisions. Confirmability was supported by independent coding, consensus discussion, and reflexive review of interpretations. Transferability was addressed by describing the study setting, assessment pathway, and participant roles in sufficient detail for readers to judge contextual similarity.

The interviewers had training and prior experience in qualitative methods and professional backgrounds in public health and clinical medicine. Because this familiarity could shape questioning and interpretation, interviewers used the same core topic guide, avoided leading prompts, recorded reflexive field notes, and discussed potential assumptions during analysis. The research team explicitly considered how its professional position within the health system could influence interpretation and sought to preserve the meaning of participants’ accounts in the coding and reporting.

### 2.5. Data analysis

We used a hybrid deductive–inductive thematic analysis. The interview domains provided initial sensitizing categories, while additional codes were generated inductively from participants’ accounts. Following the 6 phases of thematic analysis, 2 researchers first familiarized themselves with the transcripts and field notes, independently generated initial semantic codes, compared and consolidated the code set, grouped related codes into candidate themes, reviewed themes against the full dataset, and defined and named the final themes. Disagreements in coding or theme boundaries were resolved through discussion and consensus. The coding framework was refined iteratively as new insights emerged and was then applied across the dataset. Data management and coding were conducted manually; no dedicated qualitative-analysis software was used.

## 3. Results

A total of 13 participants representing multiple stakeholder groups were included in the study. The sample comprised 4 service users or family representatives (2 PWDs and 2 guardians), 7 assessment-center personnel spanning dossier intake/reception, medical examination, and leadership, and 2 district social-protection officials. To reduce the risk of deductive identification within small professional subgroups, participant characteristics are presented in aggregated role categories (Table 1). The analysis identified 4 major themes reflecting systemic challenges in disability assessment: administrative fragmentation and procedural burden, communication gaps and dependence on informal information networks, infrastructure constraints and system-level inefficiencies, and financial ambiguity and trust-related concerns. These themes were interconnected and collectively influenced the accessibility, efficiency, and perceived fairness of the assessment process.

**Table 1:** Aggregated role composition of participants.

Aggregated stakeholder category	Role in the assessment pathway	n
Service users and family representatives	Direct experience as a person with disability or guardian/legal representative	4
Assessment-center personnel and leaders	Dossier intake/reception, assessment coordination, medical examination, and center oversight	7
District social-protection officials	Preparation and submission of disability-assessment dossiers	2
Total		13

### 3.1. Theme 1: administrative fragmentation and procedural burden

Participants consistently highlighted the fragmented administrative structure as a major barrier affecting the efficiency of disability assessment. Responsibilities were distributed across multiple administrative levels (commune, district, and city) resulting in a multistep process that required coordination between different actors.

This fragmentation contributed to inconsistencies in document preparation and submission. In particular, commune-level staff were responsible for preparing dossiers, while district-level officials were responsible for submission, often leading to incomplete documentation due to indirect communication and lack of standardized guidance.

“The guidance is not always fully transmitted from the center to commune-level staff, so documents are sometimes incomplete.” (Reception official)

Delays in document processing were commonly reported, especially in districts with lower case volumes, where dossiers were collected and submitted infrequently.

“In districts with fewer cases, documents may only be collected every few months, which delays the process significantly.” (Reception official)

The interviews did not include administrative timestamp data; therefore, the frequency or duration of delay and compliance with statutory timelines could not be quantified. Participants described the operational consequence as postponed transfer and review of dossiers and later finalization of assessments. These accounts identify a handoff problem rather than simply an excessive number of steps: responsibility was distributed across administrative levels, but no single actor was described as owning completeness and timeliness across the full pathway. In addition, participants identified procedural redundancy as a source of inefficiency. Multiple referral steps were perceived as unnecessary, particularly when final assessment reports already contained sufficient justification.

Notably, perceptions of administrative burden varied across stakeholders. While administrative officials often considered the process manageable, PWDs and their families described it as complex and time-consuming, suggesting a mismatch between system design and user experience.

### 3.2. Theme 2: communication gaps and reliance on informal information networks

Formal communication channels, including public loudspeakers, community meetings, and official announcements, were widely used to disseminate information about disability assessment procedures. However, participants consistently reported that these methods were ineffective in ensuring understanding among PWDs and their families.

PWDs and caregivers described these communication approaches as passive and difficult to interpret, particularly in the absence of tailored or interactive guidance.

“The information is sometimes broadcast, but we do not fully understand what it means or what we need to do.” (Guardian)

As a result, informal information networks, particularly healthcare providers, became the primary source of guidance. Treating physicians played a key role in explaining procedures and advising patients on how to navigate the system.

“When we went to the hospital, the doctors guided us on what to prepare.” (Guardian)

Administrative staff also acknowledged that communication efforts were constrained by workload, as personnel were often responsible for multiple roles beyond disability assessment.

“Officials are responsible for many different tasks, so communication is not always prioritized.” (Reception official)

The communication problem was therefore intertwined with administrative fragmentation. When formal messages were generic and local officers had competing responsibilities, treating clinicians became de facto navigators. This arrangement assisted some applicants but made access to actionable information dependent on clinical contact rather than on a consistently understandable public information system.

### 3.3. Theme 3: infrastructure constraints and system-level inefficiencies

Infrastructure limitations were identified as a key factor affecting both the efficiency and comprehensiveness of the disability assessment process. Participants described the physical infrastructure of the assessment center as outdated, with limited space and insufficient supporting facilities.

“The infrastructure is quite old, and there is a need for better supporting facilities.” (Center leader)

A major operational challenge was the lack of specialized diagnostic equipment, which necessitated referrals to external hospitals for certain assessments. This reliance introduced additional steps, prolonged assessment timelines, and increased the logistical burden for PWDs.

“For specialized examinations, we still have to rely on hospitals, which delays the final assessment results.” (Medical examiner)

The apparently contrasting accounts concerned different dimensions of capacity. Participants valued the qualifications and breadth of the specialist workforce, while describing constraints in on-site equipment, examiner availability, coordination, and standardization. A distributed pool of expertise therefore strengthened technical coverage without necessarily ensuring timely or consistent delivery. The constraint was not a simple absence of qualified personnel, but a mismatch between professional expertise and the organizational and material resources needed to mobilize that expertise efficiently.

“We have specialists in many hospitals but coordinating them and ensuring consistency is challenging.” (Center leader)

Overall, these findings suggest that while professional capacity is adequate, infrastructural and organizational constraints limit system efficiency.

### 3.4. Theme 4: financial ambiguity and trust-related concerns

Financial issues emerged as a significant barrier, influencing both access to and perceptions of the disability assessment system. Although policies regarding reimbursement existed, many participants reported uncertainty about when payments were required and what costs were involved.

PWDs and their families often lacked prior information about assessment-related costs and only became aware of them upon arrival at the assessment center.

“Many people only realize they have to pay when they come here.” (Document reception official)

This lack of transparency led to mismatched expectations, particularly among individuals who assumed that undergoing assessment would automatically result in eligibility for benefits.

“Some people think that after paying for the assessment, they will receive benefits, but that is not always the case.” (Reception official)

In addition, certain specialized examinations were not covered by health insurance, resulting in additional out-of-pocket expenses. Financial ambiguity functioned as a procedural as well as an economic barrier. Participants did not only describe out-of-pocket payment; they described uncertainty before attendance and confusion between paying for an assessment and qualifying for benefits. This mismatch in expectations weakened confidence in the fairness and transparency of the process.

Across themes, the central implementation problem was misalignment at the points where users and dossiers moved between organizations. Administrative handoffs shaped whether accurate information was received; external referrals shaped time and costs; and uncertainty about costs and eligibility shaped trust. The 4 themes should therefore be interpreted as an interacting assessment pathway rather than as 4 independent deficits.

## 4. Discussion

This study indicates that implementation challenges in disability assessment at the Hanoi Medical Assessment Center were produced less by a single policy deficit than by misalignment across the assessment pathway. Administrative handoffs, communication, external diagnostic referrals, and financial uncertainty reinforced one another. When responsibility for dossier completeness was dispersed, users depended more heavily on informal guidance; when diagnostic services were external, additional time and cost were introduced; and when costs and eligibility were not clearly distinguished, these burdens were interpreted as unfairness. This interaction explains why a formally established policy framework did not necessarily translate into a predictable user experience (Table 2).

**Table 2:** Themes and analytical interpretation.

Theme	Key Findings	System-level Implication
Administrative fragmentation	Multilevel coordination → delays, redundancy	Fragmented coordination across administrative handoffs
Communication gaps	Formal information was not consistently understandable or actionable → reliance on healthcare professionals	Lack of user-centered communication strategy
Infrastructure constraints	Limited equipment → hospital dependency	Limited on-site diagnostic capacity and reliance on external facilities
Financial ambiguity	Unclear costs → mistrust, dissatisfaction	Uncertainty regarding costs and eligibility may reduce trust

The administrative findings add a facility-level perspective to earlier evidence from Vietnam. Banks et al reported that moving disability determination closer to commune-level authorities could improve geographic accessibility, while gaps in awareness, assessment criteria, and financial accessibility persisted.^[3]^ Our findings suggest that proximity at 1 stage does not remove coordination costs when dossiers still pass through commune, district, clinical, and city-level actors. The policy issue is therefore not decentralization alone, but whether responsibilities, completeness checks, escalation routes, and turnaround expectations are explicit at each handoff. Broader health-system reviews similarly show that decentralization can improve responsiveness while creating fragmentation when governance and coordination mechanisms are weak.^[10–15]^

The communication findings require a similarly nuanced interpretation. Participants in the present study described routine public announcements as difficult to understand and relied on healthcare professionals for practical guidance. By contrast, Palmer randomized study across 70 rural communes in Vietnam found that clear, standardized information on eligibility, benefit levels, and application procedures delivered repeatedly through government loudspeakers increased disability-benefit enrollment by approximately 9%.^[16]^ These findings are not necessarily contradictory. They suggest that the communication channel itself may be less important than message specificity, repetition, accessibility, and a direct invitation or route to action. For the Hanoi pathway, public communication may therefore be more effective if standardized messages are paired with written or digital guidance, a contact point for questions, and assistance with dossier preparation.^[17–19]^

The infrastructure and financial themes are consistent with qualitative evidence from other LMIC settings, where PWDs encounter interacting informational, physical, logistical, attitudinal, and affordability barriers. The meta-synthesis by Hashemi et al^[4]^ and recent qualitative studies in Uganda and Ethiopia describe inaccessible information, transport and facility constraints, limited specialized services, and direct costs as mutually reinforcing barriers.^[5,20]^ In our setting, respondents valued the qualifications of available specialists; the limitation lay in converting that expertise into timely service when equipment, scheduling, and coordination were distributed across institutions. Similarly, financial concern arose not only from the amount paid but also from uncertainty about insurance coverage, external examinations, and the distinction between paying for assessment and being found eligible for benefits.^[18,19]^

The findings support several center-level service priorities that can be tested without assuming national generalizability: a single, accessible checklist for dossier requirements; a clearly identified coordinator at each administrative handoff; standardized information that distinguishes assessment fees from benefit eligibility; advance disclosure of potential external examination costs; and formal coordination arrangements for specialist referrals. These are hypotheses for service improvement generated from stakeholder accounts, not evaluated interventions. Their feasibility and effects on processing time, user understanding, cost, and satisfaction should be assessed prospectively before wider scale-up.^[21–24]^

Several limitations constrain interpretation. First, this was a qualitative study at 1 urban center, and the organizational structure, resources, and communication environment may differ in rural provinces or less-resourced facilities. Second, only 4 participants were PWDs or guardians, whereas the remaining participants were staff, clinicians, leaders, or district officials; the analysis may therefore reflect provider and administrative perspectives more strongly than the full diversity of user experience. Third, the small role-specific groups required aggregated reporting to reduce deductive identification and limited comparison by participant characteristics. Fourth, the study did not collect administrative timestamps, fee amounts, or direct observations of the assessment pathway; it cannot quantify delays, determine statutory compliance, or verify differences between reported and actual practice. Fifth, interview data may be affected by recall and social-desirability bias, particularly among participants working within the system, and translation of illustrative quotations may have reduced linguistic nuance. Finally, thematic saturation within a focused sample does not establish transferability. The findings should be treated as context-specific explanations and service-improvement hypotheses that require examination in multicenter studies including rural and underserved settings.

## 5. Conclusion

In this qualitative study at a single urban assessment center, 13 stakeholders described 4 interconnected implementation problems: fragmented administrative handoffs, communication that was not consistently understandable or actionable, dependence on external diagnostic resources, and uncertainty about costs and benefit eligibility. These findings identify context-specific service priorities rather than the prevalence of problems nationally or the effects of particular policies. Within the Hanoi Medical Assessment Center pathway, practical responses may include clarifying responsibility at each handoff, providing accessible and standardized guidance, strengthening coordination of specialist services, and disclosing costs before assessment. Multicenter studies that include rural and under-resourced settings and incorporate administrative process data are needed to assess transferability and inform broader policy decisions.

## Author contributions

**Funding acquisition:** Dang Duc Nhu, Nguyen Trong Khoa, La Ngoc Quang, Phan Hoang Hiep.

**Investigation:** Dang Duc Nhu, Nguyen Ngoc Thanh, Nguyen Thanh Trung, Nguyen Ngoc Nghia, Phung Lam Toi, Nguyen Huynh Phuong Anh, Tran Thi Ly, Nguyen Trong Khoa, Duong Huy Luong, Tran Quoc Thang, Luong Thi Thu Ha, Phan Hoang Hiep.

**Methodology:** Dang Duc Nhu, Nguyen Ngoc Thanh, Nguyen Thanh Trung, Nguyen Ngoc Nghia, Phung Lam Toi, Nguyen Huynh Phuong Anh, Tran Thi Ly, Nguyen Trong Khoa, Duong Huy Luong, Tran Quoc Thang, Luong Thi Thu Ha, La Ngoc Quang, Phan Hoang Hiep, Tran Vuong The Vinh.

**Project administration:** Dang Duc Nhu, Nguyen Ngoc Thanh, Nguyen Thanh Trung, Nguyen Ngoc Nghia, Phung Lam Toi, Nguyen Huynh Phuong Anh, Tran Thi Ly, Tran Quoc Thang, Luong Thi Thu Ha, Phan Hoang Hiep, Tran Vuong The Vinh.

**Resources:** Dang Duc Nhu, Nguyen Thanh Trung, Tran Thi Ly, Nguyen Trong Khoa, Duong Huy Luong, La Ngoc Quang.

**Software:** Nguyen Ngoc Thanh, Nguyen Thanh Trung, La Ngoc Quang, Tran Vuong The Vinh.

**Supervision:** Dang Duc Nhu.

**Validation:** Nguyen Ngoc Thanh, Phung Lam Toi, Nguyen Huynh Phuong Anh, Tran Quoc Thang, Phan Hoang Hiep, Tran Vuong The Vinh.

**Visualization:** Nguyen Thanh Trung, Nguyen Ngoc Nghia, Nguyen Huynh Phuong Anh, Tran Thi Ly, Duong Huy Luong, La Ngoc Quang, Phan Hoang Hiep, Tran Vuong The Vinh.

**Writing** – **original draft:** Dang Duc Nhu, Nguyen Ngoc Thanh, Nguyen Thanh Trung, Nguyen Ngoc Nghia, Phung Lam Toi, Nguyen Huynh Phuong Anh, Tran Thi Ly, Nguyen Trong Khoa, Duong Huy Luong, Tran Quoc Thang, Luong Thi Thu Ha, La Ngoc Quang, Phan Hoang Hiep, Tran Vuong The Vinh.

**Writing** – **review & editing:** Dang Duc Nhu, Nguyen Ngoc Thanh, Nguyen Thanh Trung, Nguyen Ngoc Nghia, Phung Lam Toi, Nguyen Huynh Phuong Anh, Tran Thi Ly, Nguyen Trong Khoa, Duong Huy Luong, Tran Quoc Thang, Luong Thi Thu Ha, Phan Hoang Hiep, Tran Vuong The Vinh.


