# Supplementary material for: Health policy and facility factors influencing the effectiveness of medical assessments for people with disabilities in Hanoi, Vietnam: A qualitative study
Source: Medicine (Baltimore). 2026 Oct 2;105(40):e50922. doi: 10.1097/MD.0000000000050922 (PMC13633007; doi:10.1097/MD.0000000000050922)
Supplement: Supplementary file 1 [file medi-105-e50922-s001.docx]

**Guidelines for in-depth interviews with people with disabilities or their legal representatives**

1. **Introduction**
   - Briefly introduce yourself.
   - Explain the research purpose and objectives.
2. **Consent**
   - Invite the participant to take part in the study.
   - Request permission to record the interview.
3. **Interview Questions**
   - How is the medical assessment process for people with disabilities being conducted at the Hanoi Medical Assessment Center?
   - What factors do you think could improve the medical assessment process at the center? Please provide specific suggestions for improvement:
     - Guidelines and policies for people with disabilities in local communities.
     - Improved access to information on policies and procedures for medical assessments at the center.
     - Facility upgrades.
     - Establishing more locations in other regions.
     - Enhancing or increasing the expertise of medical assessment specialists.
     - Improving the attitudes of medical staff.
   - Do you think the center’s current medical assessment procedures are appropriate? If not, what changes are needed?
   - Which step of the medical assessment process do you find most challenging?

**Guidelines for in-depth interviews with social protection officials**

1. **Introduction**
   - Briefly introduce yourself.
   - Explain the research purpose and objectives.
2. **Consent**
   - Invite the participant to take part in the study.
   - Request permission to record the interview.
3. **Interview Questions**
   - What is your understanding of policies for people with disabilities? How do these policies affect the determination of disability levels and medical assessments? Are there any challenges?
   - How is the medical assessment process for people with disabilities being conducted? What challenges and advantages do you face in preparing and submitting disability assessment dossiers to the Medical Assessment Council?
   - Do you think the current medical assessment procedures are appropriate? If not, what changes are needed? Which steps require enhancement, and how?
   - What factors do you think could improve the medical assessment process at the center? Please provide specific suggestions for improvement:
     - Strengthening collaboration between the Medical Assessment Council and local social protection officials.
     - Training local social protection officials to enhance their skills in preparing disability assessment dossiers.
     - Providing better access to information on policies and procedures for people with disabilities.
     - Upgrading facilities and utilizing specialized medical equipment.
     - Enhancing the quality of medical assessment specialists.

**Guidelines for in-depth interviews with center leaders**

1. **Introduction**
   - Briefly introduce yourself.
   - Explain the research purpose and objectives.
2. **Consent**
   - Invite the participant to take part in the study.
   - Request permission to record the interview.
3. **Interview Questions**
   - What are the strengths and weaknesses of the current medical assessment process for people with disabilities at the center?
   - How well does the center adhere to the regulations and procedures for medical assessments? Which steps are most challenging, and why? What improvements are needed?
   - What factors do you think could enhance the medical assessment process at the center? Please provide specific suggestions for improvement:
     - Policies for medical assessment facilities.
     - Guidelines and policies for people with disabilities.
     - Changes in staffing structure.
     - Adjustments to employee remuneration.
     - Career development opportunities for healthcare staff (e.g., promotions, training).
     - Improving accessibility for people with disabilities.
     - Financial considerations.
   - Are there any upcoming plans for improving or developing medical assessment activities at the center?
